# Crystal structure of 4-bromo-*N*-(2-bromo-3-nitro­benz­yl)-2-nitro­naphthalen-1-amine

**DOI:** 10.1107/S160053681401719X

**Published:** 2014-08-01

**Authors:** Vijay P. Singh, Krishnan Venkateshwaran, Harkesh B. Singh, Ray J. Butcher

**Affiliations:** a Department of Chemistry, Indian Institute of Technology Bombay, Powai, Mumbai 400 076, India; b Department of Chemistry, Howard University, 525 College Street NW, Washington, DC 20059, USA

**Keywords:** crystal structure, naphthalen-1-amine, π–π inter­actions, hydrogen bonding, aryl­selenium compounds, photoluminescent seleno­spiro­cyclic compounds

## Abstract

In the title compound, C_17_H_11_Br_2_N_3_O_4_, the dihedral angle between the planes of the naphthalene system and the benzene ring is 52.86 (8)°. The nitro substituent and the attached naphthalene system are almost coplanar [dihedral angle = 5.6 (4)°], probably as a consequence of an intra­molecular N—H⋯O hydrogen bond with the amine group. The nitro substituent attached to the benzene ring is disordered over two sets of sites with occupancies of 0.694 (3) and 0.306 (3). The major component deviates significantly from the ring plane [dihedral angle = 53.6 (2)°]. In the crystal, the mol­ecules are linked into a three-dimensional array by extensive π–π inter­actions involving both the naphthalene and benzene rings [range of centroid–centroid distances = 3.5295 (16)–3.9629 (18) Å] and C—H⋯O inter­actions involving the methyl­ene H atoms and the phenyl-attached nitro group.

## Related literature   

For the role of secondary inter­actions in stabilizing organoselenium compounds, see; Singh *et al.* (2010 ▶, 2012 ▶); Mugesh & Singh (2000 ▶). For the isolation of novel photoluminescent seleno­spiro­cyclic compounds *via* inter­molecular C—C bond formation, see: Singh *et al.* (2011 ▶).
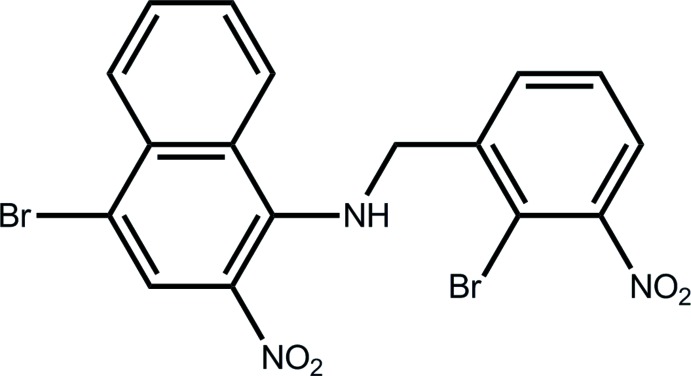


## Experimental   

### Crystal data  

C_17_H_11_Br_2_N_3_O_4_
*M* *_r_* = 481.11
Triclinic, 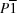

*a* = 8.3675 (4) Å
*b* = 8.5812 (5) Å
*c* = 12.2691 (5) Å
α = 76.973 (4)°
β = 81.053 (4)°
γ = 76.302 (5)°
*V* = 829.00 (8) Å^3^
*Z* = 2
Mo *K*α radiation
μ = 4.92 mm^−1^
*T* = 123 K
0.44 × 0.32 × 0.12 mm

### Data collection  

Agilent Xcalibur (Ruby, Gemini) diffractometer
Absorption correction: multi-scan (*CrysAlis PRO*; Agilent, 2012 ▶) *T*_min_ = 0.345, *T*_max_ = 1.000
12164 measured reflections
6700 independent reflections
4118 reflections with *I* > 2σ(*I*)
*R*_int_ = 0.033

### Refinement  

*R*[*F* ^2^ > 2σ(*F* ^2^)] = 0.053
*wR*(*F* ^2^) = 0.129
*S* = 1.02
6700 reflections
246 parameters
1 restraint
H atoms treated by a mixture of independent and constrained refinement
Δρ_max_ = 1.04 e Å^−3^
Δρ_min_ = −0.77 e Å^−3^

### 

Data collection: *CrysAlis PRO* (Agilent, 2012 ▶); cell refinement: *CrysAlis PRO*; data reduction: *CrysAlis PRO*; program(s) used to solve structure: *SHELXS97* (Sheldrick, 2008 ▶); program(s) used to refine structure: *SHELXL2013* (Sheldrick, 2008 ▶); molecular graphics: *SHELXTL* (Sheldrick, 2008 ▶); software used to prepare material for publication: *WinGX* (Farrugia, 2012 ▶).

## Supplementary Material

Crystal structure: contains datablock(s) I. DOI: 10.1107/S160053681401719X/tk5325sup1.cif

Structure factors: contains datablock(s) I. DOI: 10.1107/S160053681401719X/tk5325Isup2.hkl

Supporting information file. DOI: 10.1107/S160053681401719X/tk5325Isup3.cml

**Fig. 1.**
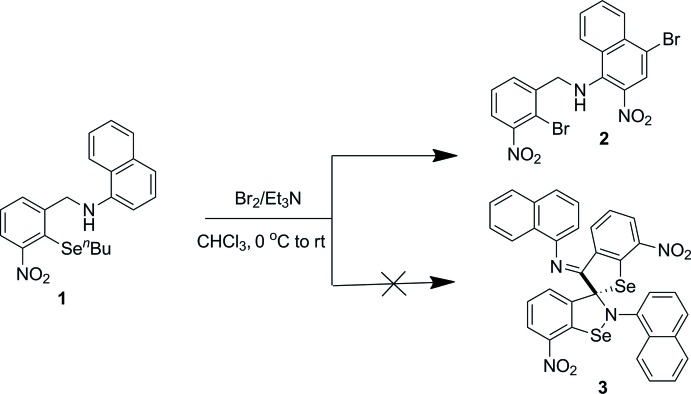
The reaction scheme.

. DOI: 10.1107/S160053681401719X/tk5325fig1.tif
The reaction scheme.

**Fig. 2.**
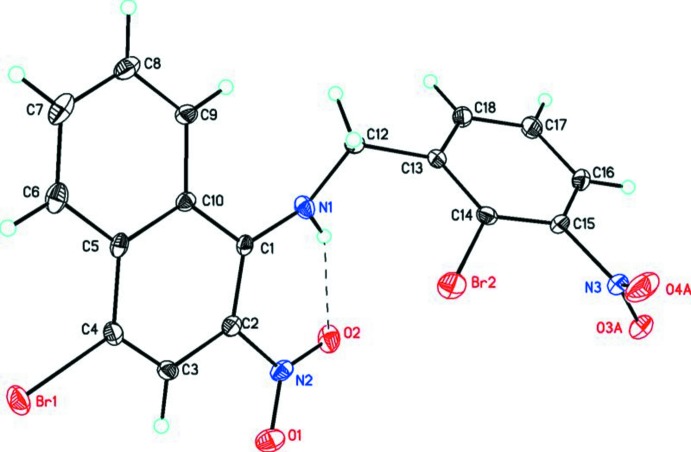
The molecular structure of C17H11Br2N3O4 showing the numbering scheme and 30% probability displacement ellipsoids and the intramolecular N—H···O hydrogen bond (shown as a dashed bond).

17 11 2 3 4 . DOI: 10.1107/S160053681401719X/tk5325fig2.tif
The mol­ecular structure of C_17_H_11_Br_2_N_3_O_4_ showing the numbering scheme and 30% probability displacement ellipsoids and the intra­molecular N—H⋯O hydrogen bond (shown as a dashed bond).

**Fig. 3.**
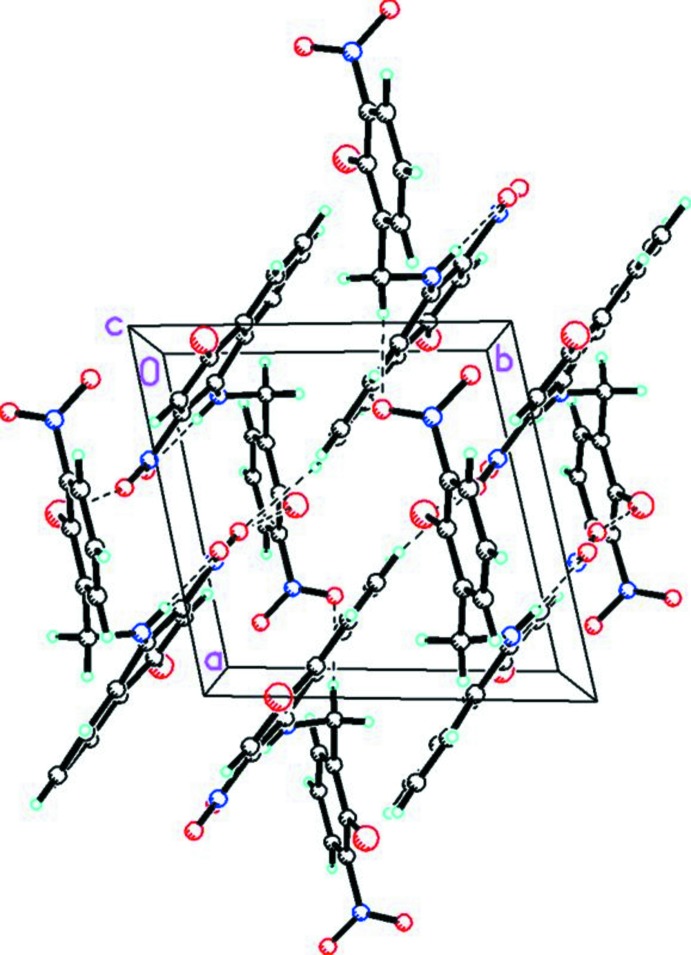
The molecular packing for C17H11Br2N3O4 viewed along the c axis showing the linking of the molecules into a three-dimensional array by π–π interactions as well as a network of C—H···O interactions (shown as dashed bonds).

17 11 2 3 4 c . DOI: 10.1107/S160053681401719X/tk5325fig3.tif
The mol­ecular packing for C_17_H_11_Br_2_N_3_O_4_ viewed along the *c* axis showing the linking of the mol­ecules into a three-dimensional array by π–π inter­actions as well as a network of C—H⋯O inter­actions (shown as dashed bonds).

CCDC reference: 1015963

Additional supporting information:  crystallographic information; 3D view; checkCIF report

## Figures and Tables

**Table 1:** Hydrogen-bond geometry (Å, °)

*D*—H⋯*A*	*D*—H	H⋯*A*	*D*⋯*A*	*D*—H⋯*A*
N1—H1*N*⋯O2	0.84 (3)	1.91 (3)	2.624 (3)	141 (3)
C12—H12*B*⋯O4*A* ^i^	0.99	2.54	3.532 (4)	177
C12—H12*B*⋯O4*B* ^i^	0.99	2.61	3.462 (8)	144

Symmetry code: (i) 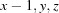.

## supplementary crystallographic information

### S1. Comment

Aryl­selenium compounds having one *ortho-*coordinating group have been widely studied as reagents in organic synthesis, gluta­thione peroxidase mimics, and precursors for the synthesis of macrocycles (Singh *et al.*, 2012; Mugesh & Singh, 2000). Introduction of a second *ortho*-coordinating group towards selenium leads to inter­esting reactivity of the selenium derivatives and isolation of unusual species (Singh *et al.*, 2010). Recently, we reported the isolation of novel photoluminescent seleno­spiro­cyclic compounds *via* inter­molecular C—C bond formation (Singh *et al.*, 2011). In continuation of this research, we attempted the synthesis of naphthyl­amine based spiro­cyclic compounds. However, the reaction led to the isolation of 4-bromo-*N*-(2-bromo-3-nitro­benzyl)-2-nitro­naphthalen-1-amine (2) instead of the desired spiro-compound (3) (Fig. 1).

In the structure of the title compound, Fig. 2, the naphthyl nitro substituent is almost coplanar with the naphthyl ring (dihedral angle = 5.6 (4)°) probably as a consequence of an intra­molecular hydrogen bond with the N—H moiety. However, the nitro substituent attached to the benzene deviates significantly from the ring plane (dihedral angle = 53.6 (2)° for the major component); this is disordered with occupancies of 0.694 (3) and 0.306 (3). The dihedral angle between the two ring systems is 52.86 (8)°. The molecules are linked into a three-dimensional array, Fig. 3, by extensive π–π inter­actions involving both the naphthyl ring (Cg1; C1, C2, C3, C4, C5, C10: Cg2; C5, C6, C7, C8, C9, C10) and benzene ring (Cg3; C13, C14, C15, C16, C17, C18), see Table 1, and, in addition, there are weak inter­molecular C—H···O inter­actions involving the methyl­ene H atoms and the benzene­nitro group, Table 2.

### S2. Experimental

Referring to Fig. 1, to a stirred solution of selenide 1 (0.400 g 1 mmol in 3 mL CHCl_3_) at 0° C, was added bromine (0.05 ml in 1 mL CHCl_3_). After 30 mins a yellow precipitate was formed. Stirring was continued for further 30 mins, Et_3_N (0.140 ml) added and the stirring continued for an additional 6 h. After completion of the reaction, the reaction mixture was poured into water and extracted with CHCl_3_ (2 × 30 mL). The combined organic layers were dried over sodium sulfate and evaporated on a rotary evaporator to get a brown solid. Yield: 0.230 g (49 %); ^1^H NMR (CDCl_3_): δ [ppm] = 7.57-7.83 (m, 6H), 8.12 (s, CH, 1H), 8.29-8.32 (d, *J* = 8.43 Hz, 1H), 8.55-8.58 (dd, *J* = 0.73, 7.33 Hz, 1H), 8.66-8.69 (dd, *J* = 0.73, 8.06 Hz, 1H). ^13^C NMR (CDCl_3_): δ [ppm] = 122.7, 123.8, 125.3, 128.1, 128.2, 128.3, 128.5, 129.2, 130.0, 132.2, 132.4, 133.2, 133.4, 135.8, 138.2, 142.5, 164.9. IR (KBr): 3455, 2924, 1666, 1510, 1374, 1296, 768, 734 cm^-1^.

### S2.1. Refinement

C-bound H atoms were placed in geometrically idealized positions and constrained to ride on their parent atoms with C—H distances of 0.95–0.99 Å, and with *U*_iso_(H) = 1.2–1.5*U*_eq_(C). The N-bound H atom was refined freely. One of the nitro groups was disordered over two conformations with occupancies of 0.694 (3) and 0.306 (4). The two conformers were constrained to have similar metrical parameters. Highest residual electron density peak; 1.02 e/Å^3^ is 0.74 A from Br2, and the deepest hole of -0.81 e/Å^3^ is 0.65 A from Br1. Twelve reflections were removed from the final refinement owing to poor agreement.

### Crystal data

**Table:**

C_17_H_11_Br_2_N_3_O_4_	*Z* = 2
*M**_r_* = 481.11	*F*(000) = 472
Triclinic, *P*1	*D*_x_ = 1.927 Mg m^−^^3^
*a* = 8.3675 (4) Å	Mo *K*α radiation, λ = 0.71073 Å
*b* = 8.5812 (5) Å	Cell parameters from 3926 reflections
*c* = 12.2691 (5) Å	θ = 5.0–34.9°
α = 76.973 (4)°	µ = 4.92 mm^−^^1^
β = 81.053 (4)°	*T* = 123 K
γ = 76.302 (5)°	Plate, orange
*V* = 829.00 (8) Å^3^	0.44 × 0.32 × 0.12 mm

### Data collection

**Table:**

Agilent Xcalibur (Ruby, Gemini) diffractometer	4118 reflections with *I* > 2σ(*I*)
Detector resolution: 10.5081 pixels mm^-1^	*R*_int_ = 0.033
ω scans	θ_max_ = 35.0°, θ_min_ = 5.0°
Absorption correction: multi-scan (*CrysAlis PRO*; Agilent, 2012)	*h* = −13→13
*T*_min_ = 0.345, *T*_max_ = 1.000	*k* = −12→13
12164 measured reflections	*l* = −19→19
6700 independent reflections	

### Refinement

**Table:**

Refinement on *F*^2^	Primary atom site location: structure-invariant direct methods
Least-squares matrix: full	Secondary atom site location: difference Fourier map
*R*[*F*^2^ > 2σ(*F*^2^)] = 0.053	Hydrogen site location: mixed
*wR*(*F*^2^) = 0.129	H atoms treated by a mixture of independent and constrained refinement
*S* = 1.02	*w* = 1/[σ^2^(*F*_o_^2^) + (0.0498*P*)^2^ + 0.3384*P*] where *P* = (*F*_o_^2^ + 2*F*_c_^2^)/3
6700 reflections	(Δ/σ)_max_ = 0.001
246 parameters	Δρ_max_ = 1.04 e Å^−^^3^
1 restraint	Δρ_min_ = −0.77 e Å^−^^3^

### Special details

**Table:**

Geometry. All e.s.d.'s (except the e.s.d. in the dihedral angle between two l.s. planes) are estimated using the full covariance matrix. The cell e.s.d.'s are taken into account individually in the estimation of e.s.d.'s in distances, angles and torsion angles; correlations between e.s.d.'s in cell parameters are only used when they are defined by crystal symmetry. An approximate (isotropic) treatment of cell e.s.d.'s is used for estimating e.s.d.'s involving l.s. planes.

### Fractional atomic coordinates and isotropic or equivalent isotropic displacement parameters (Å^2^)

**Table:**

	*x*	*y*	*z*	*U*_iso_*/*U*_eq_	Occ. (<1)
Br1	0.52119 (4)	0.68126 (4)	0.76444 (2)	0.03410 (10)	
Br2	0.98711 (4)	0.82225 (4)	0.23405 (3)	0.03346 (10)	
O1	0.9351 (3)	0.3484 (3)	0.5032 (2)	0.0374 (6)	
O2	0.8810 (3)	0.4048 (3)	0.3315 (2)	0.0346 (5)	
O3A	1.3573 (4)	0.6322 (4)	0.0114 (3)	0.0334 (7)	0.694 (3)
O4A	1.2701 (4)	0.8769 (5)	0.0461 (4)	0.0480 (10)	0.694 (3)
O3B	1.3147 (8)	0.8513 (9)	−0.0408 (7)	0.0334 (7)	0.306 (3)
O4B	1.3038 (10)	0.6768 (10)	0.1138 (8)	0.0480 (10)	0.306 (3)
N1	0.6518 (3)	0.6613 (3)	0.2639 (2)	0.0230 (5)	
H1N	0.724 (4)	0.577 (4)	0.254 (3)	0.027 (9)*	
N2	0.8518 (3)	0.4286 (3)	0.4272 (2)	0.0240 (5)	
N3	1.2471 (3)	0.7536 (3)	0.0266 (2)	0.0276 (6)	
C1	0.6183 (3)	0.6659 (3)	0.3756 (2)	0.0171 (5)	
C2	0.7114 (3)	0.5555 (3)	0.4567 (2)	0.0196 (5)	
C3	0.6804 (3)	0.5624 (3)	0.5728 (2)	0.0211 (5)	
H3A	0.7467	0.4854	0.6252	0.025*	
C4	0.5575 (3)	0.6778 (4)	0.6087 (2)	0.0216 (5)	
C5	0.4516 (3)	0.7931 (3)	0.5332 (2)	0.0183 (5)	
C6	0.3170 (3)	0.9116 (4)	0.5696 (3)	0.0264 (6)	
H6A	0.2998	0.9217	0.6463	0.032*	
C7	0.2119 (3)	1.0113 (4)	0.4962 (3)	0.0292 (7)	
H7A	0.1240	1.0917	0.5220	0.035*	
C8	0.2318 (3)	0.9967 (4)	0.3842 (3)	0.0267 (6)	
H8A	0.1552	1.0642	0.3346	0.032*	
C9	0.3622 (3)	0.8845 (3)	0.3448 (2)	0.0216 (5)	
H9A	0.3741	0.8749	0.2682	0.026*	
C10	0.4788 (3)	0.7832 (3)	0.4170 (2)	0.0171 (5)	
C12	0.6425 (3)	0.8012 (4)	0.1692 (2)	0.0224 (6)	
H12A	0.6430	0.9014	0.1961	0.027*	
H12B	0.5383	0.8185	0.1351	0.027*	
C13	0.7896 (3)	0.7678 (3)	0.0821 (2)	0.0196 (5)	
C14	0.9489 (3)	0.7762 (3)	0.0981 (2)	0.0197 (5)	
C15	1.0768 (3)	0.7462 (4)	0.0142 (2)	0.0220 (6)	
C16	1.0535 (3)	0.7066 (4)	−0.0846 (2)	0.0265 (6)	
H16A	1.1437	0.6861	−0.1406	0.032*	
C17	0.8958 (4)	0.6977 (4)	−0.1002 (2)	0.0286 (6)	
H17A	0.8765	0.6709	−0.1674	0.034*	
C18	0.7659 (3)	0.7280 (4)	−0.0171 (2)	0.0251 (6)	
H18A	0.6581	0.7213	−0.0284	0.030*	

### Atomic displacement parameters (Å^2^)

**Table:**

	*U*^11^	*U*^22^	*U*^33^	*U*^12^	*U*^13^	*U*^23^
Br1	0.03423 (17)	0.0569 (2)	0.01698 (15)	−0.01906 (15)	0.00074 (12)	−0.01145 (14)
Br2	0.03155 (17)	0.0494 (2)	0.02682 (17)	−0.01218 (14)	−0.00825 (13)	−0.01525 (14)
O1	0.0319 (11)	0.0295 (13)	0.0449 (15)	0.0057 (9)	−0.0109 (11)	−0.0035 (11)
O2	0.0336 (12)	0.0256 (12)	0.0354 (13)	0.0041 (9)	0.0090 (10)	−0.0068 (10)
O3A	0.0187 (13)	0.0379 (18)	0.0399 (18)	−0.0008 (12)	−0.0021 (12)	−0.0062 (14)
O4A	0.0293 (16)	0.042 (2)	0.077 (3)	−0.0142 (15)	−0.0194 (17)	−0.0033 (19)
O3B	0.0187 (13)	0.0379 (18)	0.0399 (18)	−0.0008 (12)	−0.0021 (12)	−0.0062 (14)
O4B	0.0293 (16)	0.042 (2)	0.077 (3)	−0.0142 (15)	−0.0194 (17)	−0.0033 (19)
N1	0.0283 (12)	0.0210 (13)	0.0178 (11)	−0.0034 (10)	0.0014 (9)	−0.0045 (9)
N2	0.0187 (10)	0.0158 (12)	0.0353 (14)	−0.0023 (9)	−0.0050 (10)	−0.0002 (10)
N3	0.0184 (11)	0.0345 (16)	0.0282 (13)	−0.0085 (11)	−0.0050 (10)	0.0023 (11)
C1	0.0144 (10)	0.0196 (13)	0.0179 (12)	−0.0063 (9)	0.0009 (9)	−0.0039 (10)
C2	0.0168 (11)	0.0173 (13)	0.0237 (13)	−0.0033 (10)	−0.0009 (10)	−0.0032 (10)
C3	0.0202 (12)	0.0237 (14)	0.0189 (13)	−0.0079 (10)	−0.0044 (10)	0.0020 (10)
C4	0.0213 (12)	0.0310 (15)	0.0162 (12)	−0.0127 (11)	−0.0005 (10)	−0.0056 (11)
C5	0.0145 (10)	0.0204 (13)	0.0220 (13)	−0.0078 (9)	0.0028 (9)	−0.0075 (10)
C6	0.0230 (13)	0.0292 (16)	0.0311 (16)	−0.0082 (11)	0.0046 (12)	−0.0163 (12)
C7	0.0185 (12)	0.0239 (16)	0.046 (2)	−0.0037 (11)	0.0035 (12)	−0.0147 (14)
C8	0.0173 (12)	0.0191 (14)	0.0401 (18)	−0.0009 (10)	−0.0033 (12)	−0.0016 (12)
C9	0.0164 (11)	0.0251 (15)	0.0229 (13)	−0.0046 (10)	−0.0052 (10)	−0.0018 (11)
C10	0.0148 (10)	0.0152 (12)	0.0206 (13)	−0.0032 (9)	−0.0023 (9)	−0.0021 (9)
C12	0.0187 (11)	0.0292 (15)	0.0179 (13)	−0.0031 (10)	−0.0026 (10)	−0.0033 (11)
C13	0.0185 (11)	0.0246 (14)	0.0149 (12)	−0.0050 (10)	−0.0023 (9)	−0.0010 (10)
C14	0.0221 (12)	0.0208 (14)	0.0167 (12)	−0.0060 (10)	−0.0062 (10)	−0.0002 (10)
C15	0.0141 (11)	0.0264 (15)	0.0244 (14)	−0.0056 (10)	−0.0039 (10)	−0.0003 (11)
C16	0.0183 (12)	0.0381 (18)	0.0205 (14)	−0.0055 (12)	0.0021 (10)	−0.0036 (12)
C17	0.0287 (14)	0.0424 (19)	0.0159 (13)	−0.0087 (13)	−0.0025 (11)	−0.0066 (12)
C18	0.0205 (12)	0.0385 (18)	0.0185 (14)	−0.0105 (12)	−0.0042 (10)	−0.0039 (12)

### Geometric parameters (Å, º)

**Table:**

Br1—C4	1.893 (3)	C6—C7	1.364 (5)
Br2—C14	1.887 (3)	C6—H6A	0.9500
O1—N2	1.231 (3)	C7—C8	1.388 (5)
O2—N2	1.214 (3)	C7—H7A	0.9500
O3A—N3	1.245 (4)	C8—C9	1.378 (4)
O4A—N3	1.199 (4)	C8—H8A	0.9500
O3B—N3	1.214 (7)	C9—C10	1.419 (4)
O4B—N3	1.224 (9)	C9—H9A	0.9500
N1—C1	1.363 (3)	C12—C13	1.517 (4)
N1—C12	1.467 (4)	C12—H12A	0.9900
N1—H1N	0.85 (3)	C12—H12B	0.9900
N2—C2	1.461 (3)	C13—C18	1.390 (4)
N3—C15	1.474 (3)	C13—C14	1.398 (4)
C1—C2	1.400 (4)	C14—C15	1.386 (4)
C1—C10	1.457 (4)	C15—C16	1.383 (4)
C2—C3	1.420 (4)	C16—C17	1.385 (4)
C3—C4	1.344 (4)	C16—H16A	0.9500
C3—H3A	0.9500	C17—C18	1.388 (4)
C4—C5	1.432 (4)	C17—H17A	0.9500
C5—C6	1.417 (4)	C18—H18A	0.9500
C5—C10	1.426 (4)		
			
C1—N1—C12	127.2 (2)	C9—C8—C7	120.2 (3)
C1—N1—H1N	111 (2)	C9—C8—H8A	119.9
C12—N1—H1N	116 (2)	C7—C8—H8A	119.9
O2—N2—O1	122.8 (3)	C8—C9—C10	121.0 (3)
O2—N2—C2	120.1 (2)	C8—C9—H9A	119.5
O1—N2—C2	117.1 (3)	C10—C9—H9A	119.5
O3B—N3—O4B	122.8 (5)	C9—C10—C5	118.2 (2)
O4A—N3—O3A	125.1 (3)	C9—C10—C1	121.3 (2)
O4A—N3—C15	118.2 (3)	C5—C10—C1	120.5 (2)
O3B—N3—C15	119.2 (4)	N1—C12—C13	109.3 (2)
O4B—N3—C15	117.1 (4)	N1—C12—H12A	109.8
O3A—N3—C15	116.7 (3)	C13—C12—H12A	109.8
N1—C1—C2	122.2 (2)	N1—C12—H12B	109.8
N1—C1—C10	121.2 (2)	C13—C12—H12B	109.8
C2—C1—C10	116.6 (2)	H12A—C12—H12B	108.3
C1—C2—C3	122.5 (2)	C18—C13—C14	118.7 (2)
C1—C2—N2	122.3 (3)	C18—C13—C12	119.2 (2)
C3—C2—N2	115.1 (3)	C14—C13—C12	122.1 (3)
C4—C3—C2	120.1 (3)	C15—C14—C13	118.8 (3)
C4—C3—H3A	120.0	C15—C14—Br2	121.3 (2)
C2—C3—H3A	120.0	C13—C14—Br2	119.9 (2)
C3—C4—C5	121.8 (3)	C16—C15—C14	122.6 (2)
C3—C4—Br1	118.3 (2)	C16—C15—N3	116.3 (2)
C5—C4—Br1	119.9 (2)	C14—C15—N3	121.1 (3)
C6—C5—C10	118.8 (3)	C15—C16—C17	118.5 (3)
C6—C5—C4	122.8 (3)	C15—C16—H16A	120.7
C10—C5—C4	118.4 (2)	C17—C16—H16A	120.7
C7—C6—C5	121.0 (3)	C16—C17—C18	119.7 (3)
C7—C6—H6A	119.5	C16—C17—H17A	120.2
C5—C6—H6A	119.5	C18—C17—H17A	120.2
C6—C7—C8	120.8 (3)	C17—C18—C13	121.7 (3)
C6—C7—H7A	119.6	C17—C18—H18A	119.2
C8—C7—H7A	119.6	C13—C18—H18A	119.2
			
C12—N1—C1—C2	−141.9 (3)	N1—C1—C10—C9	7.0 (4)
C12—N1—C1—C10	40.5 (4)	C2—C1—C10—C9	−170.8 (2)
N1—C1—C2—C3	178.1 (2)	N1—C1—C10—C5	−176.1 (2)
C10—C1—C2—C3	−4.1 (4)	C2—C1—C10—C5	6.2 (3)
N1—C1—C2—N2	0.6 (4)	C1—N1—C12—C13	138.6 (3)
C10—C1—C2—N2	178.3 (2)	N1—C12—C13—C18	105.6 (3)
O2—N2—C2—C1	−7.7 (4)	N1—C12—C13—C14	−74.5 (3)
O1—N2—C2—C1	173.1 (2)	C18—C13—C14—C15	0.6 (4)
O2—N2—C2—C3	174.6 (2)	C12—C13—C14—C15	−179.2 (3)
O1—N2—C2—C3	−4.6 (3)	C18—C13—C14—Br2	−177.7 (2)
C1—C2—C3—C4	0.2 (4)	C12—C13—C14—Br2	2.4 (4)
N2—C2—C3—C4	177.9 (2)	C13—C14—C15—C16	−0.6 (4)
C2—C3—C4—C5	1.9 (4)	Br2—C14—C15—C16	177.7 (2)
C2—C3—C4—Br1	179.86 (19)	C13—C14—C15—N3	179.9 (3)
C3—C4—C5—C6	177.4 (3)	Br2—C14—C15—N3	−1.7 (4)
Br1—C4—C5—C6	−0.6 (3)	O4A—N3—C15—C16	125.2 (4)
C3—C4—C5—C10	0.2 (4)	O3B—N3—C15—C16	60.9 (6)
Br1—C4—C5—C10	−177.74 (18)	O4B—N3—C15—C16	−129.3 (6)
C10—C5—C6—C7	2.0 (4)	O3A—N3—C15—C16	−52.1 (4)
C4—C5—C6—C7	−175.2 (3)	O4A—N3—C15—C14	−55.3 (4)
C5—C6—C7—C8	1.5 (4)	O3B—N3—C15—C14	−119.6 (6)
C6—C7—C8—C9	−2.3 (4)	O4B—N3—C15—C14	50.2 (6)
C7—C8—C9—C10	−0.5 (4)	O3A—N3—C15—C14	127.4 (3)
C8—C9—C10—C5	3.9 (4)	C14—C15—C16—C17	0.4 (5)
C8—C9—C10—C1	−179.1 (2)	N3—C15—C16—C17	179.8 (3)
C6—C5—C10—C9	−4.6 (4)	C15—C16—C17—C18	−0.1 (5)
C4—C5—C10—C9	172.7 (2)	C16—C17—C18—C13	0.2 (5)
C6—C5—C10—C1	178.4 (2)	C14—C13—C18—C17	−0.4 (4)
C4—C5—C10—C1	−4.3 (4)	C12—C13—C18—C17	179.4 (3)

### Hydrogen-bond geometry (Å, º)

**Table:**

*D*—H···*A*	*D*—H	H···*A*	*D*···*A*	*D*—H···*A*
N1—H1*N*···O2	0.84 (3)	1.91 (3)	2.624 (3)	141 (3)
C12—H12*A*···O3*B*^i^	0.99	2.57	3.117 (8)	115
C12—H12*B*···O4*A*^ii^	0.99	2.54	3.532 (4)	177
C12—H12*B*···O4*B*^ii^	0.99	2.61	3.462 (8)	144

Symmetry codes: (i) −*x*+2, −*y*+2, −*z*; (ii) *x*−1, *y*, *z*.

### π–π interactions (Å)

**Table:**

Ring 1	Ring 2	Distance	Perpedicular distance	Slippage	Symmetry	
Cg1	Cg1	3.5295 (16)	3.3867 (11)	0.94	1-x,1-y,1-z	
Cg2	Cg2	3.8868 (15)	3.3859 (12)	1.91	1-x,-y,1-z	
Cg3	Cg3	3.9629 (18)	3.5873 (12)	1.68	-x,1-y,2-z	
