# Supplementary figures and images for: Crystal structure of 4-bromo-N-(2-bromo-3-nitro­benz­yl)-2-nitro­naphthalen-1-amine
Source: Acta Crystallogr Sect E Struct Rep Online. 2014 Aug 1;70(Pt 9):o960–1. doi: 10.1107/S160053681401719X (PMC4186169; doi:10.1107/S160053681401719X)

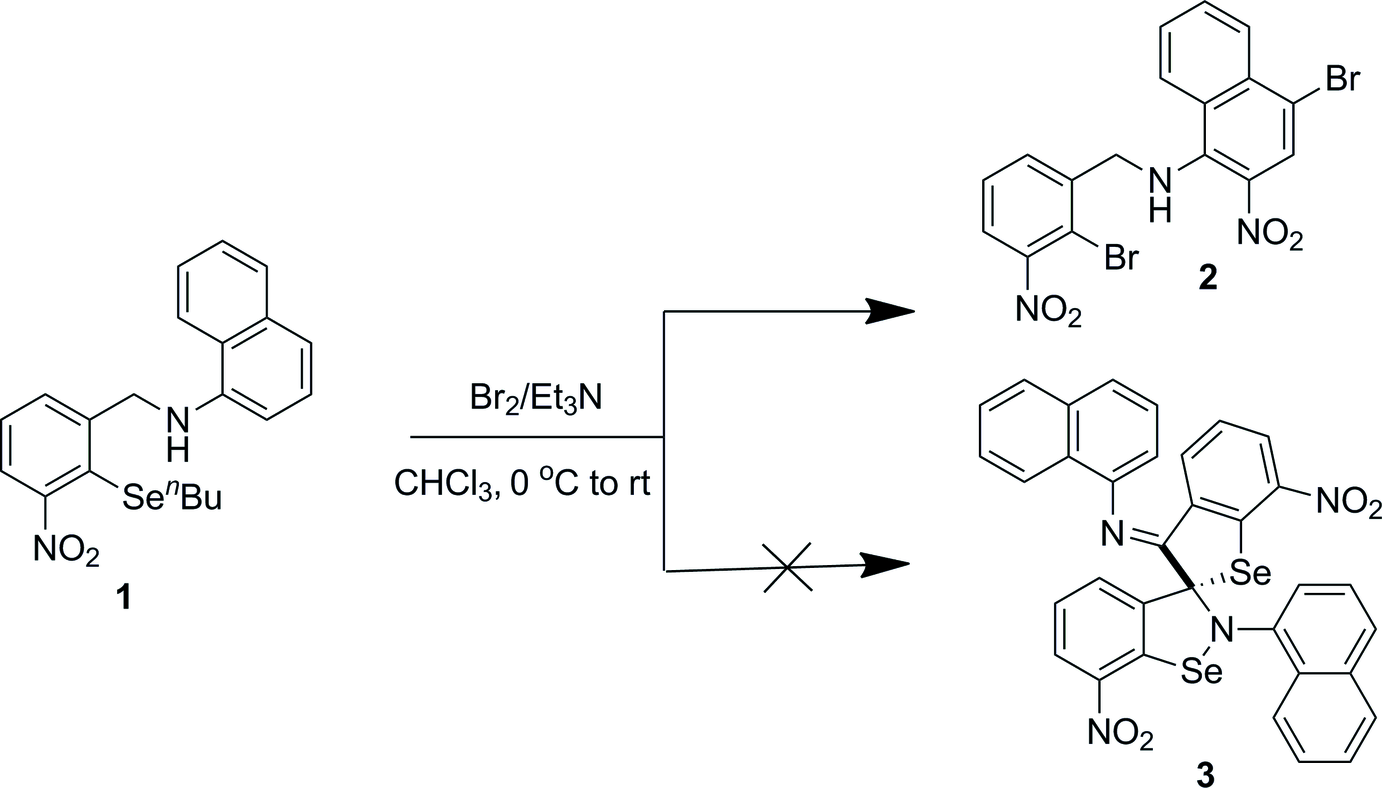

Supplement: Supplementary file 4 [file e-70-0o960-fig1.tif]

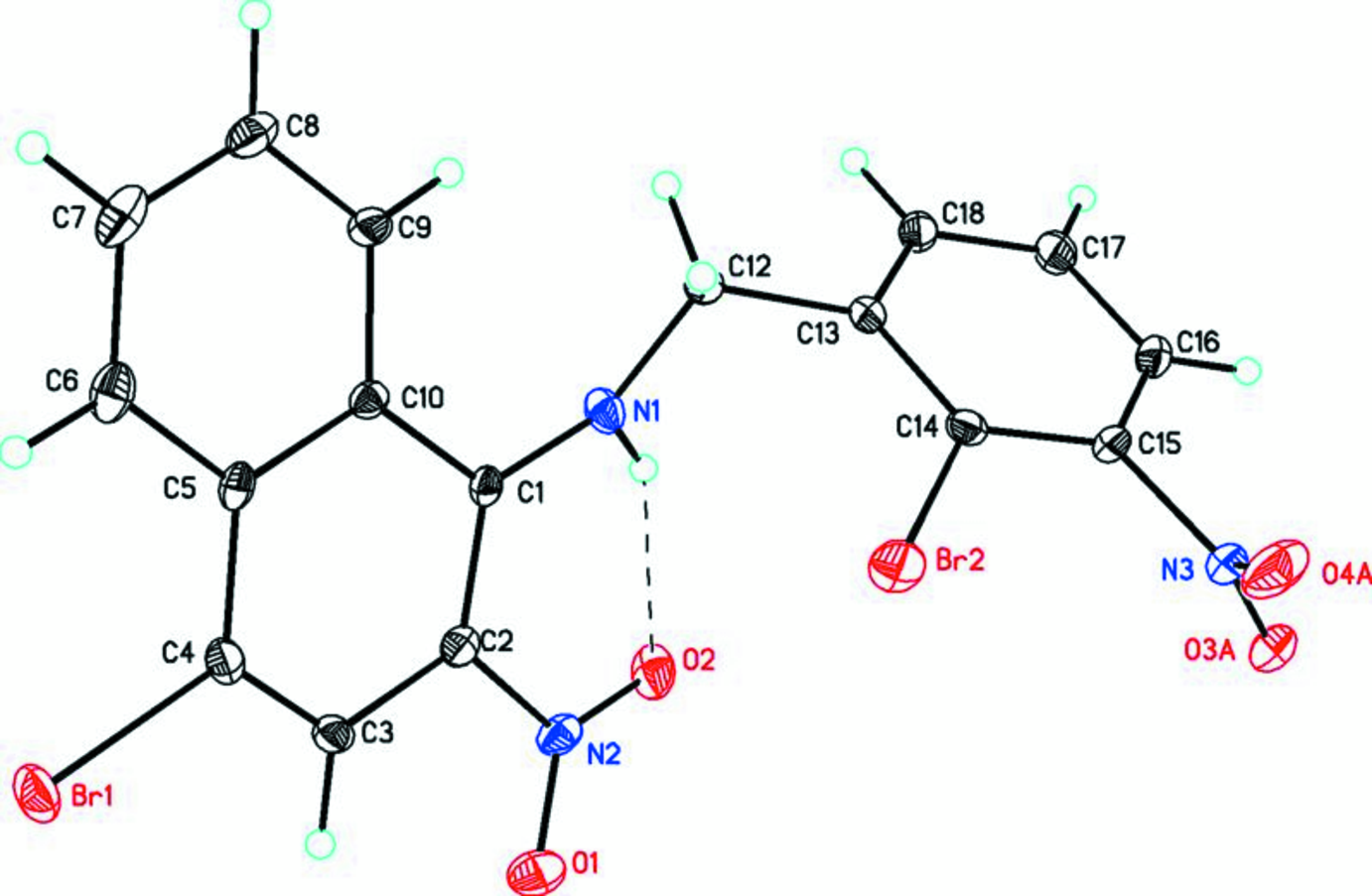

Supplement: Supplementary file 5 [file e-70-0o960-fig2.tif]

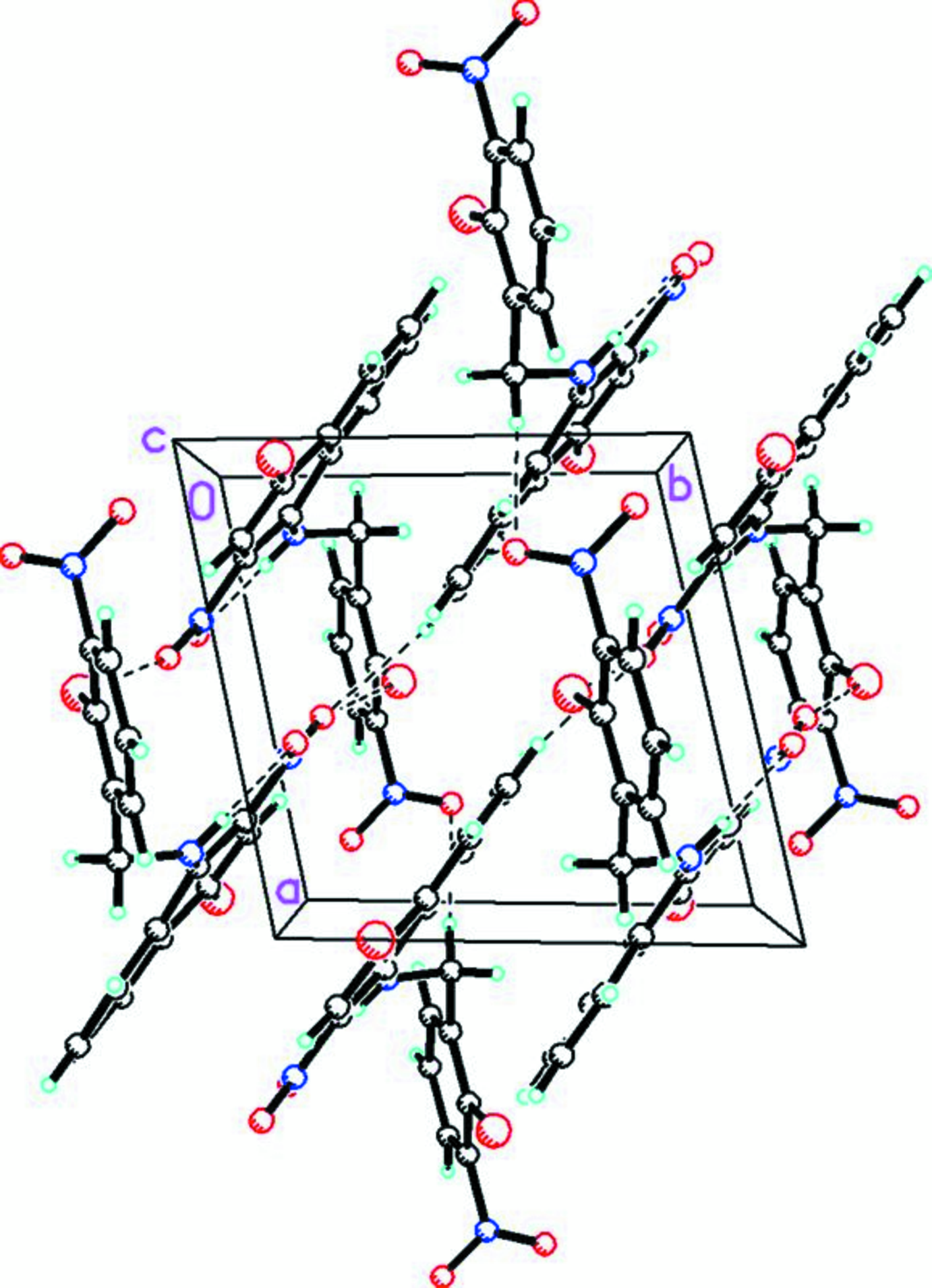

Supplement: Supplementary file 6 [file e-70-0o960-fig3.tif]
